# Skim‐sequencing for genomic selection in wheat: a comparison of marker platforms

**DOI:** 10.1002/tpg2.70197

**Published:** 2026-01-30

**Authors:** Jared L. Crain, José Crossa, Lee DeHaan, Susanne Dreisigacker, Jesse Poland, Ravi P. Singh, Paolo Vitale

**Affiliations:** ^1^ Department of Plant Pathology Kansas State University, 4024 Throckmorton Plant Sciences Center Manhattan Kansas USA; ^2^ International Maize and Wheat Improvement Center (CIMMYT), El Batan Texcoco Mexico; ^3^ The Land Institute Salina Kansas USA; ^4^ Plant Science Program, Biological and Environmental Science and Engineering Division King Abdullah University of Science and Technology Thuwal Saudi Arabia

## Abstract

The promise of genomics‐assisted breeding relies on efficient, affordable, and abundant molecular markers. Leveraging modern sequencing technology, commercial laboratory products, and open‐source software, we demonstrate how ultra‐low whole‐genome sequencing coverage (skim‐seq, 0.05–0.10x) can be a viable marker platform. The direct generation of sequence data followed by imputation provides an opportunity to implement genomic selection while being robust to future genomic (changes in reference genome) and technological (improvement in sequencing capacity) changes. We genotyped 1709 wheat (*Triticum aestivum*) lines with genotyping‐by‐sequencing (GBS), a mid‐density DArTAG single nucleotide polymorphism panel, and skim‐seq (0.07x). All skim‐seq samples were used to identify loci variants using a reference genome without the aid of any high‐coverage samples. STITCH software was used for imputation to obtain 121,437 markers. Comparing high‐confidence STITCH imputed loci (approximately 65,000 of 14 M imputed loci) to high‐coverage samples resulted in the correct imputation for more than 97.5% of the markers. Using phenotypic data, a fivefold cross validation was implemented for each marker platform. No one marker system performed the best in all test cases, with GBS often resulting in the highest correlation between observed and predicted values. The skim‐seq correlations were typically within 0.03 of GBS, suggesting skim‐seq can be a viable marker strategy for genomic prediction. As technology and computational pipelines advance, skim‐seq appears to be a promising method to bridge the gap between targeted genotyping and whole‐genome sequencing. The skim‐seq method is highly flexible and can be optimized to a variety of program needs, potentially allowing for wide adoption by the plant breeding community.

## Core Ideas

- Whole‐genome skim‐sequencing at 0.07x was used to evaluate genomic selection in a wheat breeding program.
- Genomic predictions from skim‐sequencing were equivalent to array and genotyping‐by‐sequencing predictions.
- Similar genomic regions were identified in a genome‐wide association study across marker platforms.
- Skim‐sequencing is flexible to implement while providing sequence data for future genetic studies.
- Skim‐sequencing can be tailored to leverage current and future technological advances in DNA sequencing.

## Plain Language Summary

Plant breeders rely on genomics data to enhance plant breeding efficiency. We present an ultra‐low coverage whole‐genome sequencing (skim‐seq) approach followed by imputation using open‐source software to overcome limited marker numbers and cost, limitations of routine genomic marker application in plant breeding programs. The skim‐seq method performed similar to common existing methods, including genotyping‐by‐sequencing and a mid‐density single nucleotide polymorphism panel in a set of 995 wheat lines. Comparing high‐confidence imputed data to high‐coverage samples, over 65,000 filtered high‐confidence calls of 14 M imputed loci per sample matched over 97% of the high‐coverage calls. There is considerable flexibility using skim‐seq and this approach as it does not require reference panels, prior knowledge of genomic loci, or extensive cost due to sequencing. The skim‐seq method should be applicable to a wide variety of plant breeding programs to implement genomic selection and genetic analysis.

**Abbreviations**
CIMMYT: International Maize and Wheat Improvement Center
EYT: elite yield trial
GBS: genotyping‐by‐sequencing
GS: genomic selection
HPC: high‐performance computing
MAF: minor allele frequency; PCR, polymerase chain reaction;
SNP: single nucleotide polymorphism
WGS: whole‐genome sequencing

## 1 INTRODUCTION

Genomic selection (GS) has helped accelerate plant and animal breeding. In dairy cattle, after 7 years of GS implementation, the generation interval time was reduced from nearly 7 to 2.5 years, greatly increasing genetic gain (García‐Ruiz et al., 2016). In plant breeding, GS was an important factor in the private sector developing maize (*Zea mays*, AQUAmax Dupont Pioneer) hybrids that provided 6.5% higher yield under water‐limited conditions (Cooper et al., 2014; Gaffney et al., 2015). Complementing these dynamic examples is a large body of scientific literature looking at applying GS in many animal and plant species (i.e., reviewed by Alemu et al., 2024; Dreisigacker et al., 2021, 2023; Krishnappa et al., 2021; Voss‐Fels et al., 2019).

A key premise of implementing GS is the availability of an affordable, efficient genotyping system that produces genome‐wide coverage with hundreds to thousands of markers (Heffner et al., 2009; J. Poland, Endelman, et al., 2012; Wartha & Lorenz, 2021). The application of GS in plant breeding accelerated rapidly with emerging next‐generation sequencing technologies (Mardis, 2008). While multiple marker systems have been employed to incorporate GS into plant breeding, most systems have either relied on sequence‐based genotyping or array‐based genotyping (Elbasyoni et al., 2018; J. Poland, Endelman, et al., 2012; Yu et al., 2023). Array‐based genotyping works by assaying particular sequences of DNA targets that bind to probes of oligonucleotides that are attached to known physical locations on a chip (Kockum et al., 2023; Verlouw et al., 2021). Sequencing‐based genotyping leverages next‐generation sequencing and sample preparation to sequence fragments of sample DNA using massively parallel high‐throughput sequencing (Davey et al., 2011; Elshire et al., 2011; Kockum et al., 2023; Li et al., 2010).

Specifically, within the wheat (*Triticum aestivum*) community, genotyping arrays and targeted amplicon sequencing methods have been quite common. Examples include the 35K Wheat Breeder's array (Allen et al., 2017), the Illumina 90K array (S. Wang et al., 2014), and the newer *Triticum aestivum* next generation array (Burridge et al., 2024). Along with traditional arrays, targeted amplicon sequencing, where a targeted region is amplified and then sequenced, is also commonly used in plant research and breeding communities (Zhao et al., 2024). One popular method has been DArTAG (where DArT is Diversity Array Technology, https://www.diversityarrays.com/services/targeted‐genotying/), including the One Consortium of International Agricultural Research Centers‐International Maize and Wheat Improvement Center (CIMMYT) marker platform (Garcia‐Oliveira et al., 2025). The DArTAG allows for known trait‐related markers along with genome‐wide markers in a single assay (Zhao et al., 2024). These array‐based and targeted amplicon sequencing methods have several strengths, chief among them is a high reproducibility within and across datasets and laboratories (Hong et al., 2012). Likewise, array‐based genotyping has low amounts of missing data per single nucleotide polymorphism (SNP), for example, <5% in S. Wang et al. (2014) and Burridge et al. (2024). A tradeoff between high reproducibility and low missing data is the challenge of designing arrays. Along with having costly development (Davies et al., 2016), arrays are known to have ascertainment bias (Didion et al., 2012) where genetic variation not present in the array development is not captured. In the face of potential ascertainment bias, arrays must either be designed for specific target populations or undergo continuous development of population‐specific arrays (Davies et al., 2016).

In contrast to array‐based genotyping, sequence‐based genotyping leverages the output of high‐throughput sequencing to call SNPs or other structural variations based on DNA sequences. While whole‐genome sequencing (WGS) would be the ultimate level of genome assay, WGS still remains cost prohibitive, especially for breeding programs. Even at current levels of sequencing cost, plant scientists and breeders have been able to apply novel genomic library preparations to develop effective sequence‐based genotyping. One of the most popular methods, genotyping‐by‐sequencing (GBS), reduces genome complexity through restriction enzyme digestion (Elshire et al., 2011; J. A. Poland, Brown, et al., 2012). By developing library preparation methods that selectively eliminate parts of the genome for sequencing along with highly multiplexed throughput, the cost to apply sequencing‐based genotyping approaches can be similar to or lower than array‐based options. Another significant strength of GBS is that SNP discovery can occur de novo without the need for prior genomic sequences and genomic positions (J. A. Poland & Rife, 2012).

In the absence of high genomic coverage, one of the largest challenges for sequence‐based genotyping is missing data (J. A. Poland & Rife, 2012). Given the potential application of sequence‐based data, numerous methods have been proposed to reduce genome complexity and develop reliable assays, such as exome capture and low‐coverage sequencing, which both cover the genome with markers and have sufficient sequencing depth to avoid missing data (H. Wang et al., 2023). Another significant aspect of the popular GBS method is potential licensing and proprietary issues (A. Bernardo et al., 2020). While there exist methods such as genotyping by multiplexed sequencing (Eagle et al., 2021) and multiplex restriction amplicon sequencing (A. Bernardo et al., 2020) to alleviate challenges associated with GBS, sequencing cost has continued to drop.

Regardless of genotyping platforms, imputation has become essential for genetic analysis and plant breeding. In its simplest form, imputation is inferring genotypes of loci that were not directly measured (Li et al., 2009). For both high and low levels of missing data (sequencing‐based and array‐based markers), imputation is necessary for methods that require complete data, such as developing genomic relationship matrices, and has been shown to increase the power and resolution of genome‐wide association studies (Hao et al., 2009; Quick et al., 2020). Within array‐based genotyping, imputation is often used to reduce the cost of genotyping by developing a reference panel with higher sequencing coverage, then using a sparse array containing a subset of variants within the reference panel. Finally, imputation fills in the ungenotyped loci (Howie et al., 2009; Marchini & Howie, 2010). There have been a number of imputation software tools developed to perform genomic imputation. One of the most common is Beagle, which can utilize high‐density reference panels to aid in imputation (Browning & Browning, 2016; Browning et al., 2018). Other software are specifically designed to use reference panels, such as QUILT, which uses reference panels to impute low‐coverage whole‐genome sequence data (Davies et al., 2021). While much software has been developed within human genetics, there have been efforts to develop software for imputation in species without extensive resources. The STITCH algorithm, Sequencing to Imputation Through Constructing Haplotypes, has been developed to leverage low coverage (0.15–1.70x) imputation across populations without reference panels. While STITCH requires a reference genome, imputation is performed without reference panels (Davies et al., 2016).

**Core Ideas**

- Whole‐genome skim‐sequencing at 0.07x was used to evaluate genomic selection in a wheat breeding program.
- Genomic predictions from skim‐sequencing were equivalent to array and genotyping‐by‐sequencing predictions.
- Similar genomic regions were identified in a genome‐wide association study across marker platforms.
- Skim‐sequencing is flexible to implement while providing sequence data for future genetic studies.
- Skim‐sequencing can be tailored to leverage current and future technological advances in DNA sequencing.

As genomic sequencing and output continue to become more affordable, there exists an opportunity to leverage low‐coverage sequencing data for crop improvement. Our objective was to assess the feasibility of low‐coverage skim‐sequencing (0.05–0.10x) for genomic prediction in wheat breeding. While skim‐sequencing has been previously explored for genotyping, most studies to date utilize higher coverage levels or species with smaller genomes. One of the first reports of skim‐sequencing was in rice (*Oryza sativa*) in structured recombinant inbred populations (Huang et al., 2009). In canola (*Brassica napus*), skim‐sequencing was used at levels of 0.25–5x to study structured populations, including doubled haploids and four‐parent crosses (Malmberg et al., 2018). In eggplant (*Solanum melongena*), methods to utilize 1–3x low‐coverage sequence data were developed as an alternative to high‐coverage data, providing sufficient accuracy and sensitivity when stringently filtered (Baraja‐Fonseca et al., 2025). In soybean (*Glycine max*), sequence coverage of 0.3x was deemed feasible to still retain an imputation accuracy above 97% (Happ et al., 2019). Along with applying a skim‐sequencing pipeline, we compared genomic prediction based on differing marker platforms, including a targeted amplicon sequencing, GBS, and skim‐sequencing. As the wheat genome is large (16 Gb) (Arumuganathan & Earle, 1991) and polyploid with over 85% repetitive DNA (IWGSC et al., 2018), wheat represents an excellent scenario to inform breeding and genomic activities not only in wheat but also in other crop species.

## 2 MATERIALS AND METHODS

### 2.1 Plant material

Germplasm evaluated included common wheat breeding lines in the CIMMYT elite yield trials (EYTs). The EYTs consist of approximately 1100 lines each year that are advanced from the preliminary yield trials grown during the previous year. The EYTs are phenotyped for multiple traits including grain yield, agronomic traits, disease resistance, and quality‐related traits (Juliana et al., 2020). Materials genotyped included the majority of the 2019–2020 EYT trials (*n* = 1007), a subset of lines included in the 2018–19 EYT trials (*n* = 270), which also included two high‐coverage samples (GID8240872 and GID8244129) used for imputation validation, and a subset of the 2020–2021 EYT trials (*n* = 432). For genotyping, we utilized all lines that had genomic data (*n* = 1709), and for genomic prediction, only the 2019–2020 EYT consisting of 1007 lines, was used for cross‐validation, as our goal was to test marker platform performance, and combining years could create additional genotype‐by‐environment interactions.

### 2.2 Genotyping library preparation

#### 2.2.1 Genotyping‐by‐sequencing

All EYT material was genotyped in the preliminary yield trials stage using GBS as part of the United States Agency for International Development‐funded Applied Wheat Genomics Innovation Lab, which has been described in more detail by Shrestha et al. (2025). During the preliminary yield trials, approximately 12–15,000 lines were genotyped each year, and lines that advanced would become part of the EYT trials in subsequent years. DNA extraction was completed at CIMMYT, Mexico, using fresh leaf tissue and a modified CTAB (cetyltrimethylammonium bromide) extraction (Doyle & Doyle, 1987; Dreisigacker et al., 2016). Extracted DNA was sent to Kansas State University, Manhattan, KS, where GBS libraries were prepared according to the protocol by J. A. Poland, Brown et al. (2012). Briefly, a two‐enzyme digestion was completed using *PstI* and *MspI*, and following digestion, adapters were ligated, and fragments were size selected to 170–350 bp. Single GBS libraries were composed of up to 192 samples (2 × 96‐well plates), where each sample had two unique barcodes and was sequenced on a single flowcell lane. Each library had two random blank wells, one per each 96‐well plate as a negative control and for quality control to prevent potential laboratory mix‐ups. Across the years 2017 (material used in 2018–2019 EYT) through 2019 (2020–2021 EYT), sequencing was completed on advancing Illumina platforms including, NextSeq 500, Hiseq 2500, and HiSeqX, from commercial sequencing vendors (GenomeQuebec, Novogene Corporation, and Psomagen Inc.).

#### 2.2.2 DArTAG genotyping

Genotyping using the mid‐density TaDArTAG 3.9K SNP panel (https://excellenceinbreeding.org/toolbox/services/wheat‐39k‐mid‐density‐genotyping‐services‐0) was performed by Diversity Arrays Technology. Dried leaf tissue was shipped to Australia following the service provider's recommendation, where DNA extraction, genotyping, initial quality control, and SNP calling were completed.

#### 2.2.3 Skim‐sequencing

Similar to GBS preparation, DNA was extracted and shipped to Kansas State University for skim‐sequencing library development. Even though GBS, skim‐sequencing, high‐coverage samples, and DArTAG contained the same germplasm, each genotyping activity started from different plant extractions, and a common DNA extraction for all samples followed by aliquoting DNA was not used due to the different timing and purposes of each genotyping activity.

Skim‐seq libraries, ultra‐low WGS, were prepared using a polymerase chain reaction (PCR) amplification protocol presented by Adhikari et al. (2022). Final library preparation included multiplexing four to five plates (96‐well plates) together in a single library by using dual indexes where each well had a single i7 index adapter, and each plate was barcoded with a unique i5 index adapter (Adhikari et al., 2022). Libraries were sequenced on Illumina sequencing machines at Psomagen Inc. with each library sequenced on three lanes on HiSeqX machines or two lanes on the NovaSeqX+ machines to reach a target coverage of 0.07x per sample.

#### 2.2.4 High‐coverage samples

We identified two samples, GID8240872 and GID8244129, that had high‐coverage sequencing information. Genomic DNA was extracted at CIMMYT and shipped to Kansas State University where Illumina TruSeq DNA PCR‐free library preparations were completed. The DNA extraction was from separate plants and was not from the exact plants used for GBS and skim‐seq library construction. Genomic DNA was sheared to develop libraries with a 350 bp insert. Library sequencing was completed at Psomagen Inc. on a NovaSeq 6000.

### 2.3 Bioinformatics

#### 2.3.1 GBS pipeline

Variant calling in the GBS sequence data was completed with the TASSEL GBSv2 pipeline (Glaubitz et al., 2014). For consistency with other published works using CIMMYT germplasm (Juliana et al., 2019, 2020; Shrestha et al., 2025), we followed similar bioinformatic methods, including imputation. Specifically, sequence tags from the 1709 wheat lines were aligned to the Chinese Spring IWGSC RefSev v1.0 genome (IWGSC et al., 2018) using the Bowtie2 aligner (Langmead & Salzberg, 2012) using the optimized parameters (–end‐to‐end ‐D 20 ‐R 3 ‐N 0 ‐L 10 ‐I S,1,0.25) reported by Shrestha et al. (2025) that produced the highest number of unique alignments. The final marker set was filtered for <70% missing data, biallelic SNPs with <10% heterozygosity, and a minor allele frequency (MAF) >0.05. The 14,755 markers passing the filtering threshold were imputed with Beagle v4.1 using the default settings (Browning & Browning, 2016).

#### 2.3.2 DArTAG pipeline

The results from the TaDArTAG 3.9K mid‐density panel were delivered in a flat file format that could directly be incorporated into downstream analysis. For final data use, the 3907 SNP loci were filtered for any markers missing in >30% of the samples and percent heterozygosity <20%. After filtering, the final dataset consisted of 1916 markers. As the DArTAG panel had minimal missing data, missing values were imputed with the mean imputation algorithm using *rrBLUP* (Endelman, 2011).

#### 2.3.3 Skim‐sequencing pipeline

We followed the general protocol outlined by Sthapit et al. (2025) for bioinformatic processing of the sequence files, imputation, and final marker set filtering briefly described below. Unless noted below, all software parameters were maintained at default settings. To prepare sample data for imputation, the sequenced library files were demultiplexed using the Illumina BCL software to obtain fastq files for each germplasm sample. All samples were then processed with fastp (Chen et al., 2018) to remove any adapters and low‐quality reads. Reads were then aligned to the Chinese Spring IWGSC RefSev v1.0 genome (IWGSC et al., 2018) using HISAT2 (Kim et al., 2019) allowing for up to 1000 bp gap for concordant, paired‐end alignment (‐X 1000). Sequence‐aligned files were then filtered for unique, concordant alignments with quality scores >60 using Samtools (Danecek et al., 2021), followed by sorting and writing a compressed binary alignment (BAM) file for each sample. Index files for each sample BAM file were also created using Samtools (Danecek et al., 2021).

We used STITCH v.1.6.9 software (Davies et al., 2016) to impute 14 M loci across the wheat genome with the 1712 indexed BAM files being the input into STITCH. Since all of the germplasm should be inbred and homozygous, in contrast to Sthapit et al. (2025), we used the “diploid‐inbred” option. Based on work by Shrestha et al. (2025), we used 15 as the number of ancestral haplotypes. The number of generations was set to 20, but as noted by Davies et al. (2016), STITCH is robust to parameter misspecification. Like Sthapit et al. (2025), we used parallel processing with 8 cores per chromosome and up to 240 GB of memory per job.

After STITCH imputation, we filtered for a final dataset of markers continuing the pipeline by Sthapit et al. (2025). The final set of 121,437 markers, used for all genomic predictions, was chosen as those that had an info score >0.8 (STITCH output metric that relates to the probability of the correct genotype call), MAF > 0.05, and occurred within a 5000 bp region from known gene models. We did not filter on heterozygosity as the imputation genotype calls only reflected inbred states. For genomic prediction, we used the dosage files, which are the probabilities of a genotype call rather than the qualitative allele call that only occurs when the posterior probability exceeds 90% (Davies et al., 2016).

#### 2.3.4 de novo variant identification

STITCH requires a list of positions (SNPs) to impute. SNP variants were identified by using the BCFtools software *mpileup* and *call* functions along with the Chinese Spring reference genome (IWGSC et al., 2018). To identify a set of target loci for imputation, the called variants were filtered for loci that had sequence depth ranging from 60 to 250 (approximately one half to two times the total population sequencing coverage of 120x), a MAF > 5%, and were present in at least 1% of the samples. After filtering, there was an average of 670,000 loci per chromosome (14 M total loci) that were used as targets for imputation.

#### 2.3.5 High‐coverage samples

To evaluate the accuracy of the imputed calls, the two samples (GID8240872 and GID8244129) were processed through the same bioinformatic pipeline consisting of fastp trimming, alignment to the reference genome, and filtering for unique, concordant alignment. The 121,437 loci were used as region files in BCFtools *mpileup* and *call* (Danecek et al., 2021). For comparison, genotype calls from STITCH imputed skim‐seq data of the two samples were compared for accuracy. We only compared loci where high‐coverage data resulted in a homozygous call and where STITCH imputation had imputed the low‐coverage sample with a posterior probability of 90%, that is, where a qualitative allele call was produced by the STITCH algorithm. Accuracy evaluations consisted of the number of low‐coverage STITCH imputed genotypes, only available for SNPs with a posterior probability >90%, that were the same as the high‐coverage calls divided by the total number of high‐confidence, low‐coverage STITCH imputed calls. For comparison to the high‐coverage samples, only qualitative allele calls, not dosage estimates, were used for evaluation.

### 2.4 Phenotypic data and evaluation

A total of 45 separate EYT sub‐trials (or lattices) were evaluated during 2019–2020 in a two‐replicate alpha‐lattice design, and each trial consisted of 28 breeding lines plus two check varieties included for each unique environment (Juliana et al., 2020). We followed the phenotyping and analyses for grain yield, agronomic traits, and disease resistance as described by CIMMYT (Juliana et al., 2020; Singh et al., 2022), where a mixed linear model (ASREML, Gilmour et al., 2015) was used to estimate a best linear unbiased estimate for each collected observation. Broad sense heritability for the yield trials was estimated to range from 0.56 to 0.88 indicating quality field trials. Our 2019–2020 EYT dataset included 15 variables, of which six were grain yield in selection environments, seven were disease scores and ratings, and the final two were heading date and plant maturity. Selection environments comprised a combination of different sowing dates, systems, and irrigation regimes. For detailed descriptions of these experimental setups, refer to Juliana et al. (2020). Grain yield was collected at the CIMMYT station in Ciudad Obregon, Mexico, while disease scores were evaluated in Ciudad Obregon, El Batan, Toluca, and Agua Fria (Mexico), Njoro (Kenya), and Ludhiana (India). The selection environments and planting combinations relate to the different mega‐environments that CIMMYT targets for breeding efforts (Braun et al., 1996).

### 2.5 Genomic prediction and genome‐wide association models

To assess how different genomic marker platforms would work for genomic prediction, we implemented a fivefold cross‐validation using the 2019–2020 EYT (*n* = 995 after filtering for samples with DArTAG, GBS, and skim‐seq data) germplasm along with the collected phenotypic data. We assigned a random group (1–5) to each genotype within the cross‐validation set. This grouping was maintained for each marker dataset so that the training and prediction sets were always the same for each trait with only the molecular markers differing. For each iteration, 1–5, onefold of the data was masked while the remaining four folds trained the model. The masked fold was predicted, followed by iterating until all folds had been predicted. As GBS and skim‐seq both had more markers than the DArTAG panel, we also implemented a reduced marker set by randomly drawing 1916 markers and repeating the prediction pipeline. All GS models were implemented using genomic best linear unbiased prediction in the *rrBLUP* package (Endelman, 2011). The correlation between the predicted BLUPs and masked phenotypic values was evaluated for each different marker set. A 95% confidence interval was created using the *psychometric* package (Fletcher, 2023). To further assess prediction accuracy, we repeated the process of randomly selecting 80% of the samples to train the model and the remaining 20% of the data were masked and predicted. This process was carried out 1000 times for each marker set and trait. A standard error of the mean was developed for each trait and marker combination. As much of the CIMMYT material has been utilized in existing publication, we explored the yellow (stripe) rust (*Puccinia striiformis* West.) dataset in more detail. In Juliana et al. (2020), a strong marker trait association was found on chromosome 2B. We utilized the EYT yellow rust rating in Ludhiana, India, and completed a genome‐wide association study using *rrBLUP* (Endelman, 2011) for all three marker platforms. All statistical analysis and graphics were completed in R software (R Core Team, 2022) with packages including *CMplot* (Yin et al., 2021) and *PerformanceAnalytics* (Peterson & Carl, 2024).

## 3 RESULTS

### 3.1 Marker system results

**FIGURE 1:**
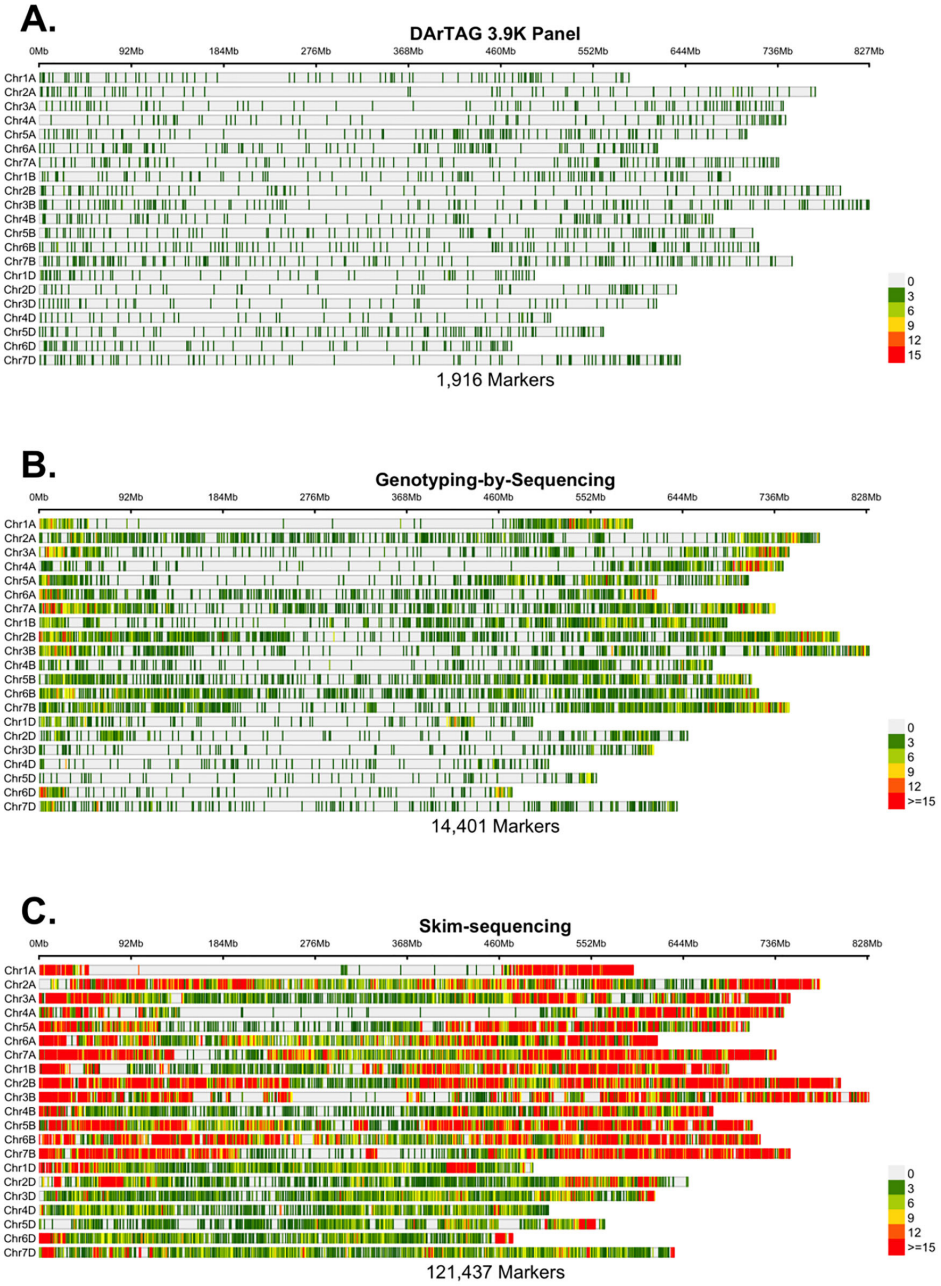
Distribution of genotyped loci across the wheat genome for (A) DArTAG panel of 1916 markers, (B) 14,401 genotyping‐by‐sequencing (GBS) markers, and (C) 121,437 skim‐sequencing markers. Plots are shaded by the number of markers per megabase.

We implemented three separate genotyping systems in 1712 CIMMYT wheat lines, of which 995 were part of the 2019–2020 EYT trial. Our overall objective was to compare skim‐seq, where we used ultra‐low coverage WGS of 0.07x, to existing marker platforms. As an initial assessment of marker platforms, we evaluated the filtered, final marker set distribution over the wheat genome (Figure 1). The sheer number of skim‐seq markers (121,437) resulted in overall superior genome coverage compared to GBS and DArTAG markers. We do note that the skim‐seq markers had large gaps, particularly in chromosomes 1A and 4A. In these regions, the DArTAG markers were more evenly distributed likely from targeting known genomic regions compared to de novo SNP discovery. Subsequent results use markers that are presented in Figure 1; however, for future application it may be possible to adjust filtering criteria to identify additional markers in marker poor regions. The number of variants ranged from a genome average of 1 variant per 8.8, 1.1, and 0.1 Mb for DArTAG, GBS, and skim‐seq, respectively.

**FIGURE 2:**
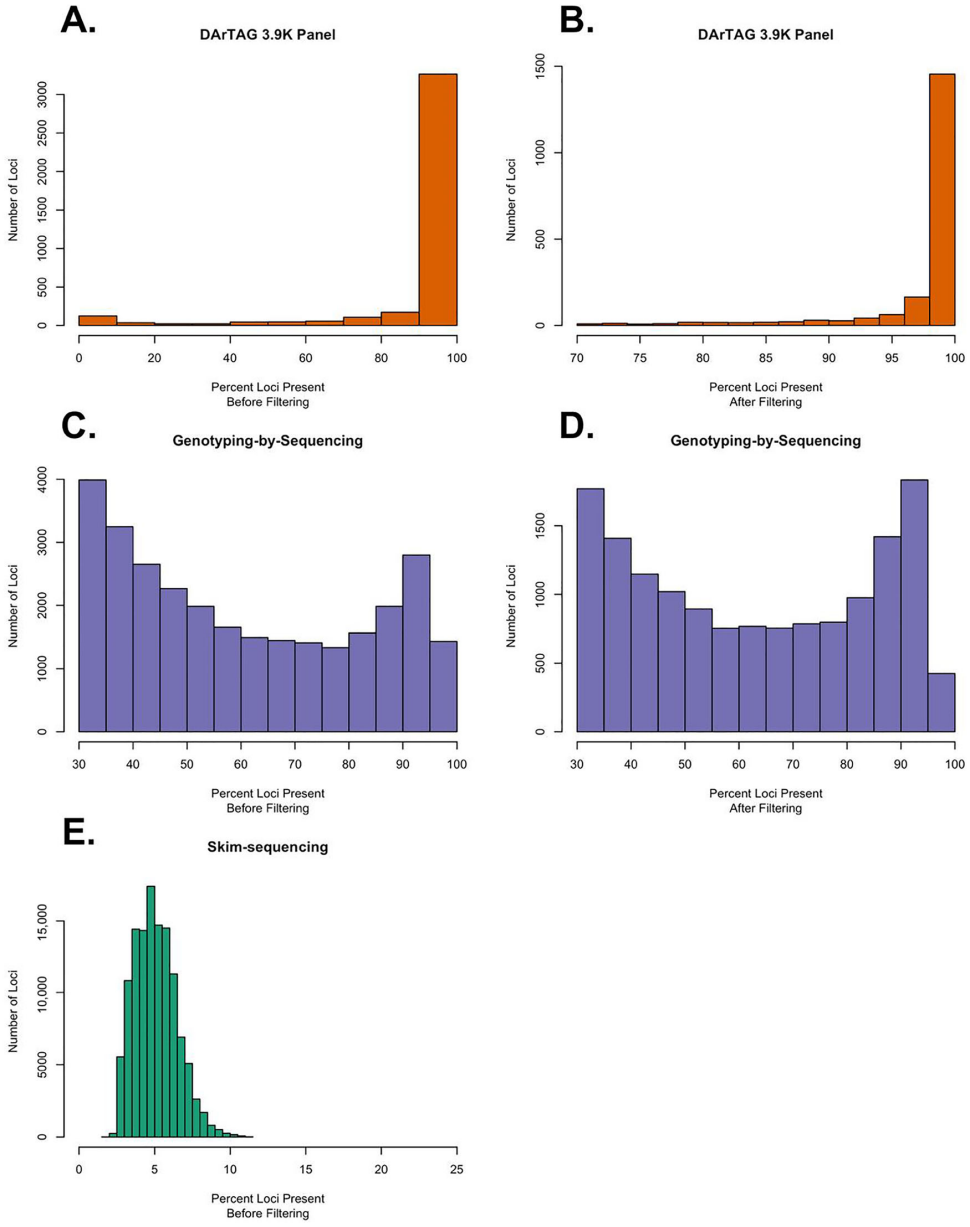
Distribution of observed (percent present) markers before and after filtering for 1709 wheat lines from the International Maize and Wheat Improvement Center for three different genotyping platforms. Panels include (A) DArTAG before filtering, (B) DArTAG after filtering, (C) genotyping‐by‐sequencing (GBS) before filtering, (D) GBS after filtering, and (E) skim‐sequencing (skim‐seq) befor filtering for the final dataset. Skim‐seq after filtering is not presented as all skim‐seq data are imputed from ultra‐low (0.07x) whole‐genome sequencing.

**FIGURE 3:**
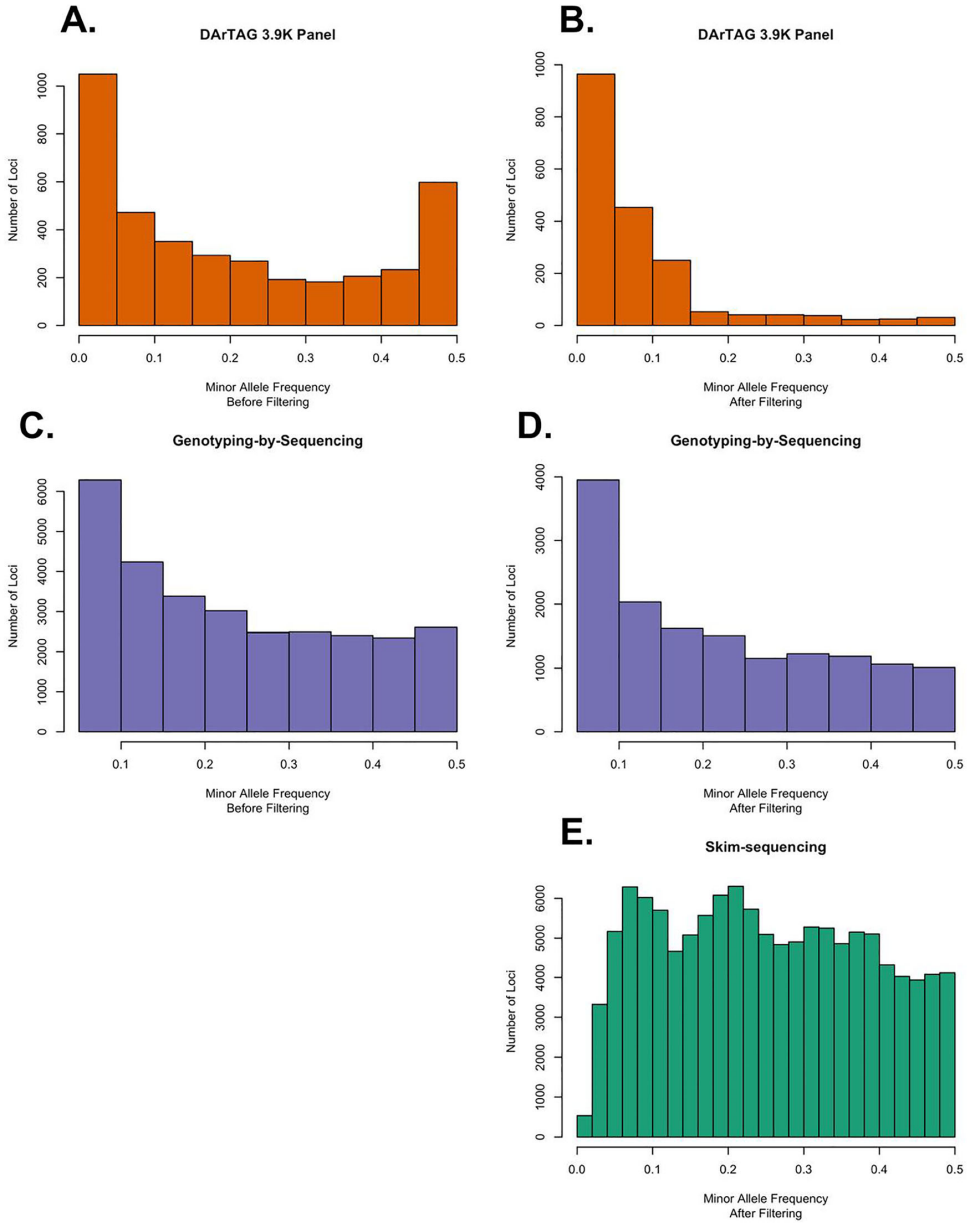
Minor allele frequency distribution before and after filtering for 1709 wheat lines from the International Maize and Wheat Improvement Center for three different genotyping platforms. Panels include (A) DArTAG before filtering, (B) DArTAG after filtering, (C) genotyping‐by‐sequencing (GBS) before filtering, (D) GBS after filtering, (E) skim‐sequencing (skim‐seq) after filtering for the final dataset. Skim‐seq before filtering is not presented as all skim‐seq data are imputed from ultra‐low (0.07x) whole‐genome sequencing.

Beyond marker distribution, we also evaluated the amount of data present in each marker system before imputation (Figure 2), as well as key population parameters including MAF (Figure 3) and percent heterozygosity (Figure S1) in the initial and filtered datasets. The results were in line with expectations that DArTAG had the most called data, while skim‐seq at 0.07x coverage had the least called data per loci (Figure 2). Our target coverage of 0.07x coverage aligned well with observations where a mean of 5.1% of loci were observed (Figure 2E). The number of reads per loci was very low, with observed loci having a mean read depth of 1.2 in the skim‐seq data. In contrast, the GBS data had a mean read depth of 5.1 suggesting greater certainty in SNPs called by GBS.

Filtering maintained the approximate distribution of data (Figures 2 and 3) except for removing a heavy tail of high MAF in the DArTAG data (Figure 3). The heavy tail was removed using only a percent heterozygosity filter as the DArTAG data were not filtered specifically on MAF. The skim‐seq data profile of MAF showed a more uniform distribution of SNPs across the range of 0–0.5, but a peak still occurred at lower MAF. As the skim‐seq data were imputed, and there was minimal coverage, certain skim‐seq distributions were not possible to calculate. Specifically, there were no heterozygosity data as STITCH only output homozygous calls in the “diploid‐inbred” mode and the minimal read depth would make genotype calls unreliable. Nevertheless, evaluating percent heterozygosity in DArTAG and GBS data revealed a large proportion of loci with heterozygosity above 20% (Figure S1), and suggesting that filters targeting low heterozygosity were appropriate for inbred germplasm.

#### 3.1.1 Comparison of skim‐sequencing to high coverage

Within the panel of 1709 genotyped lines, GID8240872 and GID8244129 were sequenced at 8.5 and 13.6x, respectively through other projects. Using the 121,437 markers imputed by STITCH, the loci for these sites were obtained from the high coverage data. Evaluation was only carried out for SNPs imputed with a posterior probability >90%, which resulted in a qualitative SNP call, as below a 90% posterior probability, STITCH would only output a quantitative dosage value. When comparing STITCH genotype calls to the sequenced high‐coverage data, we evaluated 67,139 and 66,813 loci for the respective samples out of 14 M imputed loci. STITCH correctly imputed 97.5% and 97.7% of the loci for each sample, indicating that the imputation was highly accurate even with extremely low (GID8240872 = 0.063x and GID8244129 = 0.070x) skim‐seq data followed by imputation without reference panels or other high‐coverage data.

### 3.2 Genomic prediction

To evaluate genomic predictions using skim‐seq data, we implemented a fivefold cross validation in the 2019–2020 CIMMYT EYT material. There were a total of 15 different observation sets that included six grain yields in different selection environments and seven disease comparisons providing a dataset that would be typical of plant breeding programs. Across the 15 datasets, no one marker platform was consistently the best (Table 1); however, there were general trends observed within the data. Typically, GBS markers performed the best, with 10 of 15 datasets resulting in a statistically significant difference between GBS and DArTAG markers. Where DArTAG markers outperformed GBS (two datasets), the results were not statistically different. Genomic predictions made with skim‐seq markers were typically lower than GBS. (Table 1).

**TABLE 1:** Correlation between masked genomic predicted value and phenotypic best linear unbiased estimator for 995 wheat lines from the 2019 to 2020 elite yield trial at the International Maize and Wheat Improvement Center for three different genotyping marker platforms: DArTAG, genotyping‐by‐sequencing (GBS), and ultra‐low coverage whole‐genome sequencing (skim‐seq).

Phenotypic trait ID	DArTAG	GBS	Skim‐seq
Days to heading, bed planting, 5 Irrigation, Ciudad Obregon, Mexico	0.38a	0.53b	0.52b
Fusarium head blight index, El Batan, Mexico	0.23a	0.33b	0.34b
Grain yield bed planting, 2 Irrigation, Ciudad Obregon, Mexico	0.47a	0.52b	0.50ab
Grain yield, bed planting, 5 Irrigation, Ciudad Obregon, Mexico	0.48a	0.53b	0.50b
Grain yield, bed planting, heat, Ciudad Obregon, Mexico	0.32a	0.40b	0.36ab
Grain yield, bed planting, late heat, Ciudad Obregon, Mexico	0.36a	0.54c	0.45b
Grain yield, bed plant, drought, Ciudad Obregon, Mexico	0.45a	0.52b	0.49ab
Grain yield, flat planting, 5 Irrigation, Ciudad Obregon, Mexico	0.49a	0.49a	0.44b
Plant maturity, bed planting, 5 Irrigation, Ciudad Obregon, Mexico	0.44b	0.55a	0.54a
Spot blotch, Agua Fria, Mexico	0.59a	0.62a	0.59a
Stem rust, Njoro, Kenya	0.62b	0.69a	0.63b
Septoria tritici blight, Toluca, Mexico	0.42a	0.52b	0.45a
Yellow rust, Njoro, Kenya	0.51a	0.50a	0.50a
Yellow rust, Ludhiana, India	0.52a	0.52a	0.48a
Yellow rust, Toluca, Mexico	0.37a	0.36a	0.34a

*Note*: Letters indicate groupings based on statistical differences outside of the 95% correlation confidence interval.

**FIGURE 4:**
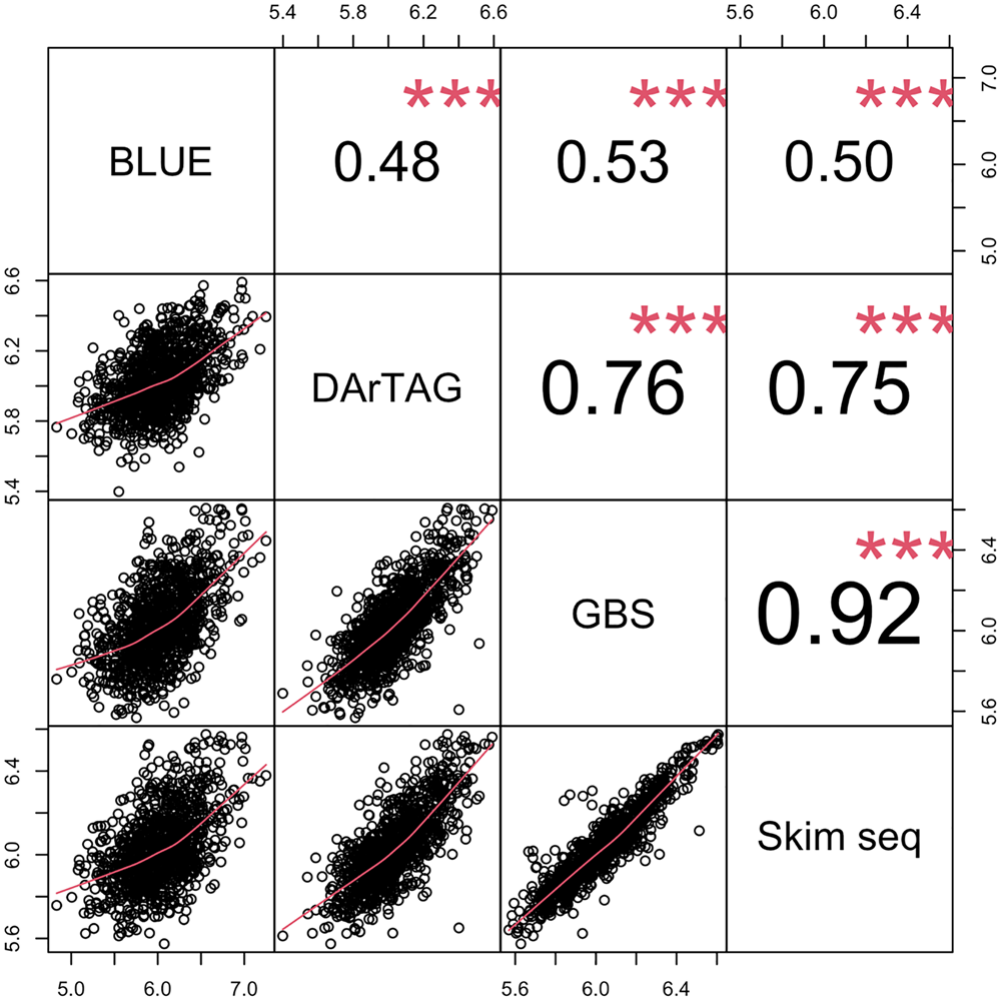
Panel plot showing the relationship between grain yield and predicted genomic values using a fivefold cross‐validation strategy in the 2019–2020 elite yield trial bed planting with five irrigations at Ciudad Obregon, Mexico. Columns include the best linear unbiased estimator (BLUE) for grain yield, predictions made by DArTAG markers, genotyping‐by‐sequencing (GBS) markers, and skim‐sequencing markers (skim‐seq). ***p < 0.001.

As the DArTAG panel had much lower marker number (n = 1916) than GBS or skim‐seq, we also reduced the GBS and skim‐seq marker set by randomly drawing 1916 markers. When performing genomic prediction using these random sets, the correlation for both GBS and skim‐seq was reduced (0.025), yet similar general trends were maintained (Table S1). To further assess the difference between marker types, we implemented 1000 iterations of randomly training 80% of the data followed by predicting the remaining 20%. While these results offered more precision around the mean prediction accuracy, the overall trends compared to the fivefold cross‐validation were maintained where no one marker system performed the best for all traits (Table S2). Our results indicate that the sequence‐based markers typically performed better than targeted amplicon‐based markers. However, we realize this difference, while often statistically significant, likely has minor implications for practical breeding (Figure 4). All marker systems resulted in high correlations between platforms, suggesting an equivalency between marker systems.

### 3.3 Genome‐wide association study

**FIGURE 5:**
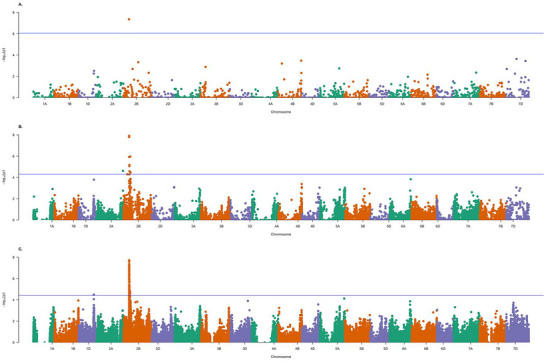
Manhattan plot for genome‐wide association study for yellow rust phenotype collected at Ludhiana, India, for the International Maize and Wheat Improvement Center 2019–2020 elite yield trial data. Panels indicate different marker platforms for the same yellow rust phenotype, including (A) 1916 DArTAG markers, (B) 14,401 genotyping‐by‐sequencing markers, and (C) 121,437 low‐coverage skim‐sequencing markers.

To further validate the observed genomic prediction trends, we conducted a genome‐wide association study of yellow rust collected in Ludhiana, India. Previous analysis by Juliana et al. (2019) showed a strong marker trait association spanning 6.2 Mb (155155706–161419325) on chromosome 2B. Using the same phenotypic dataset and only changing the marker dataset from DArTAG, GBS, and skim‐seq, we observed a significant marker trait association for all marker sets on chromosome 2B (Figure 5). Within the DArTAG panel, only one marker was significant on chromosome 2B at position 155142247. Likewise, GBS identified the same marker position and a 2.1 Mb region associated with six markers (2B 155142247–157266347). Within the skim‐seq dataset, which had the most markers, a strong 2.7 Mb peak ranging from 154931981 to 157649149 was identified and included 15 significant markers. The MAF was calculated for the most significant marker in each analysis and was 0.11 (DArTAG), 0.17 (GBS), and 0.17 (skim‐seq) suggesting that each marker platform was assessing the population similarly. These results indicate that imputed, low‐coverage data can be successfully used for additional genomic studies.

## 4 DISCUSSION

### 4.1 Application of skim‐sequencing

We evaluated an emerging genotyping system that uses commercial library preparation and open‐source software for bioinformatic processing. Notably, the system scales to breeding‐size populations as skim‐sequencing can pool over 3000 uniquely barcoded samples into a single DNA sequencing library (Adhikari et al., 2022). Using only the wheat reference genome, we used the total population coverage to identify variants and impute a final dataset on samples with an average of 0.07x coverage. While there are previous examples of low‐coverage imputation, most rely on deeper sequencing of reference samples (panels) for imputation (Browning & Browning, 2016; Davies et al., 2021; Jordan et al., 2022; Rubinacci et al., 2021; Sthapit et al., 2025). Compared to many previously published reports on skim‐sequencing (Baraja‐Fonseca et al., 2025; Huang et al., 2009; Kumar et al., 2021; Malmberg et al., 2018), we utilized material from an entire breeding program representing a wider range of diversity showing that skim‐seq can be used beyond well‐structured populations. Even considering the entire CIMMYT bread wheat breeding program, the diversity is likely restricted (Shrestha et al., 2025), which could influence our results; however, for routine GS application where cost is paramount, our results are promising for plant breeding programs. These results are further bolstered by the successful application of STITCH into routine breeding application at 0.05x coverage of the outcrossing, heterozygous intermediate wheatgrass (*Thinopyrum intermedium*) program where diversity and linkage blocks would be expected to be more diverse than wheat (Sthapit et al., 2025).

We used the framework for the skim‐seq pipeline as proposed by Sthapit et al. (2025) to evaluate GS predictions across a wheat breeding program. Within the pipeline, there are multiple parameter adjustments that can be made. We tested the potential of skim‐seq to perform similarly to GBS or targeted amplicon genotyping for GS. Both GBS and targeted amplicon genotyping have been evaluated in comparison multiple times with researchers typically concluding that the methods provide equivalent results in terms of genomic information (Bajgain et al., 2016; Elbasyoni et al., 2018; J. Poland, Endelman, et al., 2012). Our starting parameters from variant identification to STITCH imputation were estimates based on prior work (Sthapit et al., 2025). For implementation in a new species or breeding program, parameter tuning and optimization may help improve accuracy by identifying high‐confidence loci. While STITCH has been shown to be robust to parameter settings like the number of generations and ancestral haplotype (Davies et al., 2016), similar to GS models (Heslot et al., 2012), parameter optimization could result in improving efficiency simply through computational processes.

Along with parameter optimization for accuracy, time optimization is critical for plant breeding programs. For practical implementation, estimates of 1 week or less of processing time for genomic predictions have been published (Bassi et al., 2016; Crain et al., 2021). The current bioinformatics pipeline can be run from demultiplexing samples to imputed genotypes in less than 5 days using parallel array jobs operating on each chromosome independently (Sthapit et al., 2025). Overall, the genotyping and imputation pipeline appears robust and offers sufficient flexibility for applications involving diverse crops and breeding programs.

### 4.2 Comparison of marker methods

Our results indicate that skim‐seq can be an effective marker platform for plant breeders. While skim‐seq leverages advancing sequencing technology for effective genotyping in a breeding program, it also produces low‐coverage WGS that can be used for genetic studies. Even in a large genome like wheat, sequencing cost for 0.1x coverage (5.3 M reads) per sample can now be completed for less than $5 per sample. Within a plant breeding program, DNA extraction and library preparation, likewise, cost approximately $5 per sample resulting in a total of $10 per sample excluding labor. While every program will be different, from our experience, two technicians processing 20,000 samples per year can result in a labor cost of $10 per sample. To achieve these cost levels, large sample numbers, which are typical of plant breeding programs utilizing GS, are required. While we targeted 0.07x, lower target coverage levels (0.05x) have been reported in intermediate wheatgrass, an outcrossing, heterozygous, hexaploid species, with successful results (Sthapit et al., 2025). Both advancing technology and sequencing coverage optimization could be used to further refine cost‐effective protocols using skim‐seq. Regardless, even in the large wheat genome, sequencing is no longer the cost‐constrained step that has historically been a limiting factor (Bentley, 2006). At $10, the skim‐seq is more affordable per marker than the $13 (https://excellenceinbreeding.org/toolbox/services/mid‐density‐genotyping‐service) for the TaDArTag 3.9K panel, while providing tens of thousands more markers. Compared to GBS, skim‐seq likewise results in more potential loci at a similar or reduced cost without any licensing issues (A. Bernardo et al., 2020; J. A. Poland, Endelman, et al., 2012). We envision that skim‐seq and imputation could be applied to a wide variety of crops, while we have used wheat as a model, for crops with smaller genomes such as rice (*Oryza sativa*, 430 Mb genome; Kurata, 2002), the same cost of sequencing would result in over 3.5x coverage, or pearl millet (*Cenchrus americanus*, 1.79 Gb genome; Varshney et al., 2017) at 0.9x coverage. At these and higher coverage levels, there exists a variety of software that could be utilized for genotype imputation (Davies et al., 2021; Rhee et al., 2016; Triay et al., 2025; Watowich et al., 2023).

Effective implementation of any marker system is essential for breeding programs. While we have evaluated three diverse sequence‐based genotyping systems, each system has its unique benefits and drawbacks (Table 2). From an applied application of GS in plant breeding, our results show that any of the tested marker systems would result in equivalent application. While skim‐seq will leverage continual advances in DNA sequencing technology, we recognize that the application of skim‐seq or GBS requires a high level of bioinformatic expertise and access to computational infrastructure. While all GS predictions were completed on a local laptop, the DArTAG service did not require any high‐performance computing (HPC). Both the GBS markers and skim‐seq markers required access to HPC resources and large storage volumes for sequence data to demultiplex, call SNPs, impute, and filter results to a minimal subset appropriate for laptop computing. For resource‐limited programs, cloud infrastructure and data storage may be a viable option to overcome these challenges. If skim‐seq becomes more popular, the amount of software and pipelines dedicated to the approach will likely increase, similar to how there are well‐defined GBS pipelines (Glaubitz et al., 2014) making the method more tractable to a wider audience.

**TABLE 2:** A comparison of advantages and disadvantages of targeted amplicon or array‐based genotyping (DArTAG example), genotyping‐by‐sequencing, and ultra‐low coverage whole‐genome sequencing (skim‐seq) for plant breeding programs.

	Marker type	Targeted amplicon/array‐based	Sequence‐based	Sequence‐based
Utilization	Example	DArTAG	GBS	Skim‐seq
Data	Missing data	Minimal	0%–70%^a^	90%+^a^
	Data confidence	High	Medium^a^	Variable^a^
	Amount of data	Variable based on array^a^	Medium^a^	High^a^
Application	Ease of processing	High	Medium	Low
	Computational requirements	Laptop	HPC	HPC
	Tools available	Commercial	Commercial—Cottage Industry	Bespoke pipelines
Reusability	Combine multiple datasets	Variable based on datasets	Simple, update pipeline	Simple, update pipeline
	Identify new variants	Difficult, update array	Simple, sequence and update pipeline	Simple, sequence and update pipeline
Cost	Infrastructure	Minimal	HPC	HPC
	Personnel	Minimal training	Some computational experience	Advanced computational skills
	Per sample	$12–$250^a^ (Bassi et al., 2016)	$10–$40 + licensing^a^ (Bassi et al., 2016; A. Bernardo et al., 2020)	$10 dependent on sequencing coverage^a^

Abbreviation: GBS, genotyping‐by‐sequence; HPC, high‐performance computing; Skim‐seq, Skin‐sequencing.

^a^ Areas where project design could influence outcome based on array selection, amount of sequencing, and filtering criteria.

Contrasting the ease of compiling a usable marker set, skim‐seq and GBS both allow for larger levels of data use and reuse (J. A. Poland & Rife, 2012; Scheben et al., 2017). The input fastq files are the starting point for many other bioinformatic software and analyses and allow quick combination of datasets compared to the challenges of combining different arrays (Johnson et al., 2013). Likewise, the ability to update genomic positions or utilize a different reference genome is simply a matter of updating pipeline scripts and rerunning analysis. Both GBS and skim‐seq can be optimized to run in limited (less than 1 week) timeframes even with up to 20,000 samples (Crain et al., 2021; Sthapit et al., 2025). The ability to identify de novo variants is a strength of both GBS (J. A. Poland & Rife, 2012) and skim‐seq because new and diverse samples can be added to the analysis. Genotyping based on arrays or targeted amplicons will be limited to the material that was used to develop the array and cannot be easily expanded to diverse material without updating or recreating the array targets (Burridge et al., 2024) or having additional reference panels (Davies et al., 2021). Finally, we note that skim‐seq provides a broader representation of the entire genome and is not limited to restriction enzyme sites or constrained by potential licensing issues (A. Bernardo et al., 2020) that could increase cost beyond program resources.

### 4.3 Future applications of skim‐seq

Anticipating further sequencing, computational, hardware, and software advancements, skim‐seq provides a usable format (fastq sequence file) of future‐proof data. Work by Adhikari et al. (2022) showed that low levels of skim‐seq (0.01x coverage) could be used to track introgression of wild material and identify chromosomal contribution of parents in segregating populations. Within plant breeding, accurate genotyping of introgressions or known alleles is essential for efficient marker‐assisted selection strategies. Based on the large number of markers successfully imputed, skim‐seq could be directly used to track introgression within germplasm while providing whole genome information. We hypothesize that for introgression projects, only sufficient sequencing coverage of the starting wild material, similar to variant ascertainment presented, could result in successful allele tracking.

Outside of breeding activities, the application of skim‐seq provides large datasets with which to analyze genetic architecture. Our evaluation of yellow rust was based on prior observations of strong genetic signal for this trait and location. All three marker platforms identified the same genomic regions as Juliana et al. (2019). In addition to CIMMYT material, other researchers have identified this region (Agenbag et al., 2014; Athiyannan et al., 2022; Beukert et al., 2020; Rollar et al., 2021; Shahinnia et al., 2022) with yellow rust resistance. Athiyannan et al. (2022) identified this location as the disease resistance gene *Yr27*. Based on imputation accuracy and as demonstrated with yellow rust, we believe that genome‐wide association studies should be feasible with skim‐seq data.

For a plant breeding program, large datasets across thousands of individuals could provide opportunities for population analysis and allele tracking. Modification of pool‐sequencing (Schlötterer et al., 2014) of entire populations, such as in silico pooling of uniquely barcoded samples representing phenotypic differences (Crain et al., 2023), could be an attractive method for learning about allele frequencies and their changes within breeding programs. As programs develop multiple years of resources, time‐series analysis from pool‐sequencing and modified bioinformatics could provide real‐time insight within breeding programs (Taus et al., 2017).

Within this work, we used STITCH imputation software (Davies et al., 2016), but the skim‐seq data could be subjected to other imputation software. For example, a practical haplotype graph (Jensen et al., 2020; Jordan et al., 2022) could be bolstered by low‐coverage data to identify new variants or used to impute the low‐coverage data. Even with low‐coverage data, where confidence in any single loci call may be low, our results show that low‐coverage data are accurate enough to generate genomic relationship matrices for GS. Given that we found over 65k markers of high‐confidence calls, another option is to complete a two‐stage imputation where the high‐confidence calls from the imputed low‐coverage could be the input into other imputation programs like Beagle (Teng et al., 2022).

## 5 CONCLUSIONS

Breeding programs are inherently data‐centric. Since the 1980s, molecular markers have offered the promise to revolutionize plant breeding (R. Bernardo, 2008, 2016). Within plant breeding where large quantities of germplasm are easy and affordable to produce, methods in molecular breeding such as GS (Meuwissen et al., 2001) outpaced technological advances to practically implement within plant breeding. Today, technological advances are positioning plant breeding to reap the rewards of data‐driven discovery, and skim‐seq provides a step change from capturing targeted genomic locations, whether through restriction enzymes, known arrays, or targeted capture, to assaying entire genomes. The flexibility of the method along with existing tools should allow skim‐seq to become common place in plant breeding programs across species. Whether a species has had large resource investment for reference genomes or pangenomes (i.e., wheat [IWGSC et al., 2018; Walkowiak et al., 2020]) or is just starting along a molecular pathway, skim‐seq should be applicable to advance breeding and genetic gains.

## AUTHOR CONTRIBUTIONS

**Jared L. Crain**: Conceptualization; formal analysis; methodology; writing—original draft; writing—review and editing. **José Crossa**: Resources; writing—review and editing. **Lee DeHaan**: Funding acquisition; writing—review and editing. **Susanne Dreisigacker**: Resources; writing—review and editing. **Jesse Poland**: Funding acquisition; resources; writing—review and editing. **Ravi P. Singh**: Resources; writing—review and editing. **Paolo Vitale**: Resources; writing—review and editing.

## CONFLICT OF INTEREST STATEMENT

The authors declare no conflicts of interest.

## Supporting information



Supplementary Material

## Data Availability

All scripts and data files, including DArTAG markers, to implement the entire analysis have been placed in a Zenodo digital repository at https://doi.org/10.5281/zenodo.15360500. All GBS sequencing data have previously been uploaded to NCBI Sequence Read Archive (SRA) (https://www.ncbi.nlm.nih.gov/bioproject/) as part of the umbrella BioProject PRJNA901997, subproject PRJNA498085. Skim‐Sequence data has been uploaded to the NCBI SRA as PRJNA1139475.The high‐coverage sequence data has been uploaded to the NCBI SRA as PRJNA1263701.
