# Supplementary material for: Skim‐sequencing for genomic selection in wheat: a comparison of marker platforms
Source: Plant Genome. 2026 Jan 30;19(1):e70197. doi: 10.1002/tpg2.70197 (PMC12871549; doi:10.1002/tpg2.70197)
Supplement: Supplementary file 1 [file TPG2-19-e70197-s001.docx]

Supplemental Material

Supplementary Table 1. Correlation between masked genomic predicted value and phenotypic best linear unbiased estimator for 995 wheat lines from the 2019-2020 Elite Yield Trial at the International Maize and Wheat Improvement Center for three different genotyping marker platforms DArTAG, genotyping-by-sequencing (GBS), and ultra-low coverage whole-genome sequencing (skim-seq). Random GBS and Skim-seq are equal number (n=1,916) of markers as DArTAG randomly sampled from total marker number.

| **Phenotypic Trait ID** | **DArTAG** | **GBS** | **GBS Random** | **Skim seq** | **Skim seq Random** |
| --- | --- | --- | --- | --- | --- |
| Days to Heading, Bed Planting, 5 Irrigation, Ciudad Obregon, Mexico | 0.38 | 0.53 | 0.48 | 0.52 | 0.47 |
| Fusarium Head Blight Index, El Batan, Mexico | 0.23 | 0.33 | 0.34 | 0.34 | 0.32 |
| Grain Yield Bed Planting, 2 Irrigation, Ciudad Obregon, Mexico | 0.47 | 0.52 | 0.51 | 0.50 | 0.47 |
| Grain Yield, Bed Planting, 5 Irrigation, Ciudad Obregon, Mexico | 0.48 | 0.53 | 0.52 | 0.50 | 0.46 |
| Grain Yield, Bed Planting, Heat, Ciudad Obregon, Mexico | 0.32 | 0.40 | 0.38 | 0.36 | 0.35 |
| Grain Yield, Bed Planting, Late Heat, Ciudad Obregon, Mexico | 0.36 | 0.54 | 0.46 | 0.45 | 0.42 |
| Grain Yield, Bed Plant, Drought, Ciudad Obregon, Mexico | 0.45 | 0.52 | 0.50 | 0.49 | 0.44 |
| Grain Yield, Flat Planting, 5 Irrigation, Ciudad Obregon, Mexico | 0.49 | 0.49 | 0.47 | 0.44 | 0.40 |
| Plant Maturity, Bed Planting 5 Irrigation, Ciudad Obregon, Mexico | 0.44 | 0.55 | 0.53 | 0.54 | 0.50 |
| Spot Blotch, Agua Fria, Mexico | 0.59 | 0.62 | 0.60 | 0.59 | 0.54 |
| Stem Rust, Njoro, Kenya | 0.62 | 0.69 | 0.66 | 0.63 | 0.58 |
| Septoria tritici Blight, Toluca, Mexico | 0.42 | 0.52 | 0.49 | 0.45 | 0.42 |
| Yellow Rust, Njoro, Kenya | 0.51 | 0.50 | 0.47 | 0.50 | 0.49 |
| Yellow Rust, Ludhiana, India | 0.52 | 0.52 | 0.50 | 0.48 | 0.46 |
| Yellow Rust, Toluca, Mexico | 0.37 | 0.36 | 0.34 | 0.34 | 0.35 |

**Supplementary Table 2.** Mean correlation of 1,000 iterations of randomly sampling 80% of the genotypes and predicting the remaining 20%. Correlation is the predicted value compared to the masked value for 15 different traits for 995 wheat lines from the 2019-2020 Elite Yield Trials at the International Maize and Wheat Improvement Center. Sampling was repeated for three marker platforms, DArTAG, genotyping-by-sequencing (GBS), and ultra-low coverage whole-genome sequencing (skim-seq). Across 1,000 iterations, the standard error of the mean was less than 0.002 for all trait and marker combinations. The 95% confidence interval (CI) for the mean is included.

| Phenotypic Trait ID | DArTAG | | GBS | | Skim_seq | |
| --- | --- | --- | --- | --- | --- | --- |
|  | Mean | CI | Mean | CI | Mean | CI |
| Days to Heading, Bed Planting, 5 Irrigation, Ciudad Obregon, Mexico | 0.367 | ± 0.003 | 0.521 | ± 0.003 | 0.515 | ± 0.003 |
| Fusarium Head Blight Index, El Batan, Mexico | 0.259 | ± 0.004 | 0.362 | ± 0.003 | 0.353 | ± 0.003 |
| Grain Yield Bed Planting, 2 Irrigation, Ciudad Obregon, Mexico | 0.486 | ± 0.003 | 0.540 | ± 0.003 | 0.510 | ± 0.003 |
| Grain Yield, Bed Planting, 5 Irrigation, Ciudad Obregon, Mexico | 0.498 | ± 0.003 | 0.533 | ± 0.003 | 0.506 | ± 0.003 |
| Grain Yield, Bed Planting, Heat, Ciudad Obregon, Mexico | 0.300 | ± 0.004 | 0.393 | ± 0.003 | 0.356 | ± 0.003 |
| Grain Yield, Bed Planting, Late Heat, Ciudad Obregon, Mexico | 0.355 | ± 0.004 | 0.513 | ± 0.003 | 0.448 | ± 0.003 |
| Grain Yield, Bed Plant, Drought, Ciudad Obregon, Mexico | 0.447 | ± 0.003 | 0.520 | ± 0.003 | 0.479 | ± 0.003 |
| Grain Yield, Flat Planting, 5 Irrigation, Ciudad Obregon, Mexico | 0.506 | ± 0.003 | 0.513 | ± 0.003 | 0.458 | ± 0.003 |
| Plant Maturity, Bed Planting 5 Irrigation, Ciudad Obregon, Mexico | 0.443 | ± 0.003 | 0.550 | ± 0.003 | 0.549 | ± 0.003 |
| *Spot Blotch,* Agua Fria, Mexico | 0.572 | ± 0.003 | 0.627 | ± 0.002 | 0.592 | ± 0.003 |
| Stem Rust, Njoro, Kenya | 0.637 | ± 0.002 | 0.686 | ± 0.002 | 0.629 | ± 0.003 |
| *Septoria tritici Blight,* Toluca, Mexico | 0.436 | ± 0.003 | 0.524 | ± 0.003 | 0.458 | ± 0.003 |
| Yellow Rust, Njoro, Kenya | 0.511 | ± 0.003 | 0.509 | ± 0.003 | 0.514 | ± 0.003 |
| Yellow Rust, Ludhiana, India | 0.508 | ± 0.003 | 0.515 | ± 0.003 | 0.475 | ± 0.003 |
| Yellow Rust, Toluca, Mexico | 0.372 | ± 0.004 | 0.353 | ± 0.004 | 0.335 | ± 0.004 |

**Supplementary Figure 1**. Percent heterozygosity of DArTAG markers and genotyping-by-sequencing (GBS) markers before and after filtering observed in 1,709 wheat lines from the International Maize and Wheat Improvement Center. Panels are (A) DArTAG before filtering, (B) DArTAG after filtering, (C) GBS before filtering, and (D) GBS after filtering.
